# Multitemporal Terrestrial Laser Scanning for Marble Extraction Assessment in an Underground Quarry of the Apuan Alps (Italy)

**DOI:** 10.3390/s19030450

**Published:** 2019-01-22

**Authors:** Silvia Di Bartolo, Riccardo Salvini

**Affiliations:** Department of Environment, Earth and Physical Sciences and Centre of GeoTechnologies CGT, University of Siena, Via Vetri Vecchi 34, 52027 San Giovanni Valdarno (AR), Italy

**Keywords:** Terrestrial Laser Scanning, 3D point cloud, multitemporal change detection, volume computation, marble quarrying

## Abstract

This article focuses on the use of Terrestrial Laser Scanning (TLS) for change detection analysis of multitemporal point clouds datasets. Two topographic surveys were carried out during the years 2016 and 2017 in an underground marble quarry of the Apuan Alps (Italy) combining TLS with Global Navigation Satellite System (GNSS) and Total Station (TS) studies. Multitemporal 3D point clouds were processed and compared with the aim of identifying areas subjected to significant material extraction. Point clouds representing changed areas were converted into triangular meshes in order to compute the volume of extracted material over one year of quarrying activities. General purpose of this work is to show a valid method to examine the morphological changes due to raw material extraction with the focus of highlighting benefits, accuracies and drawbacks. The purpose of the executed survey was that of supporting the planning of quarrying activities in respect of regional rules, safety and commercial reasons.

## 1. Introduction

The use of Terrestrial Laser Scanning (TLS) data for the determination of morphological variations is gaining increasing interest in geological surveying and monitoring. TLS is widely used, among the others, in rock slope studies [1,2,3], river science [4,5,6] and coastal cliff monitoring [7,8]. In recent years, several authors have experimented with the use of TLS both in underground and in open pit quarrying activities [9,10,11,12,13,14]. TLS is based on the acquisition of a high-density point cloud in a very short period of time basically using different techniques: pulse-based (time-of-flight) or phase-based. In the first method, the scanner measures the time a laser pulse takes to travel from a source to a reflective surface and back again (round trip); from the knowledge of the time, given that the velocity is equal to the speed of light, the distance between the source and the target can be computed. The working principle of the second type is based on the modulation in amplitude of the emitted (incoherent) light; the scattered reflection from a surface is collected and the system measures the phase difference between the sent and received waveforms, hence the delay time. Typical phase-based scanners modulate their signal using sinusoidal modulation, amplitude-based (AM) or frequency- based (FM) modulation, and pseudo-noise or polarization modulation [15,16]. In this study, the TLS survey was carried out using a pulse-based static device which measures the range between source and target through an infrared light. After scanning, with the goal of generating realistic virtual reality 3D models and orthophotos, photographs may also be collected by using a digital external camera. The repeated application of TLS to the same area (i.e., multitemporality) allows the comparison between corresponding 3D models. Conventional techniques for topographic change detection, even from TLS data, rely on the comparison of Digital Elevation Models (DEMs) to produce a new DEM called “DEM of Differences” (DoD) [17,18]. The DoD technique, unfortunately, cannot operate properly on complex 3D environments since the DEMs, needing a plane of reference (typically on the XY coordinates plane), can describe with difficulties vertical and rough surfaces. DEMs are interpolated and uncertainties may be introduced on the grid elevation when the point clouds represent either vertical or protruding fronts [19,20]. New algorithms allow for direct comparison between 3D point clouds without gridding or meshing the point clouds and several experiences have been testified in recent manuscripts dealing with multitemporal comparison [21,22,23,24,25,26,27,28,29]. Multi scale Model to Model Comparison (M3C2) plugin operates in 3D so that it alleviates a key limitation of the DoD technique [30]. For this study M3C2 method was used to compute distances between the point clouds and to conduct change detection analysis. Then, Poisson Surface Reconstruction algorithm proposed by Kazhdan et al. [31] was used to create high detail triangular meshes from point clouds representing the excavated areas. Volume of single extraction area was calculated and computation error estimated in a precautionary approach basing on multitemporal point clouds misalignments and meshing uncertainties.

## 2. Study Area and Geological Setting

**Figure 1:**
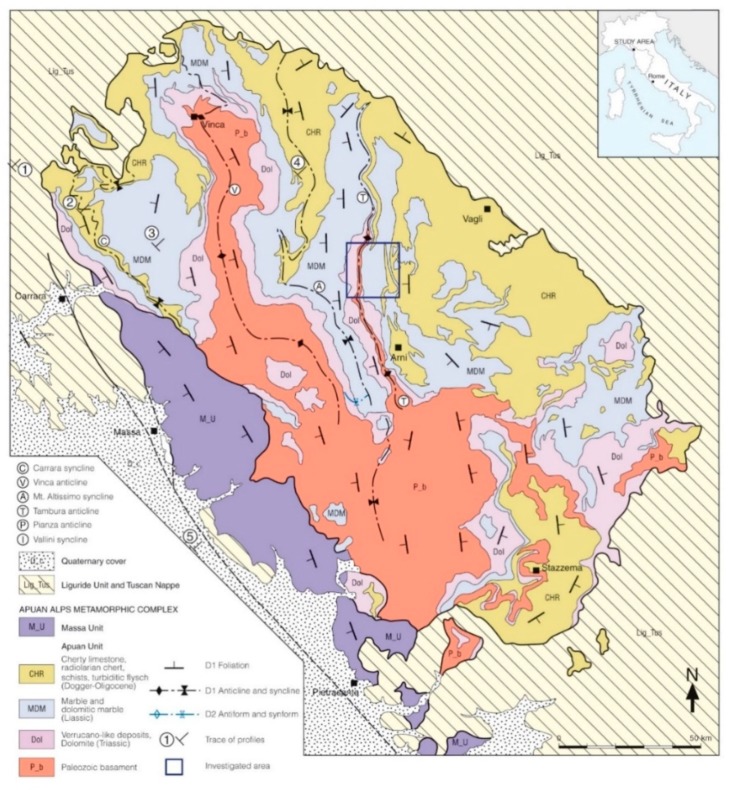
Geological sketch map of the Apuan Alps. The location of the quarry under study is highlighted by a blue rectangle (modified after [35]).

The investigated underground marble quarry, known as “Piastreta”, is located in the area of the Sella Mount, in the Apuan Alps (Province of Massa-Carrara, Italy) at 1580 m a.s.l. The Apuan Alps metamorphic complex (Figure 1) is composed of two main units, the Massa Unit and the Apuan Unit. Deep levels of the inner Northern Apennines are here exposed [32,33,34] since it represents the largest tectonic window of the belt. The metamorphic complex tectonically holds up the unmetamorphic formations of the Tuscan Nappe which is in turn overthrusted by the Liguride Units.

**Figure 2:**
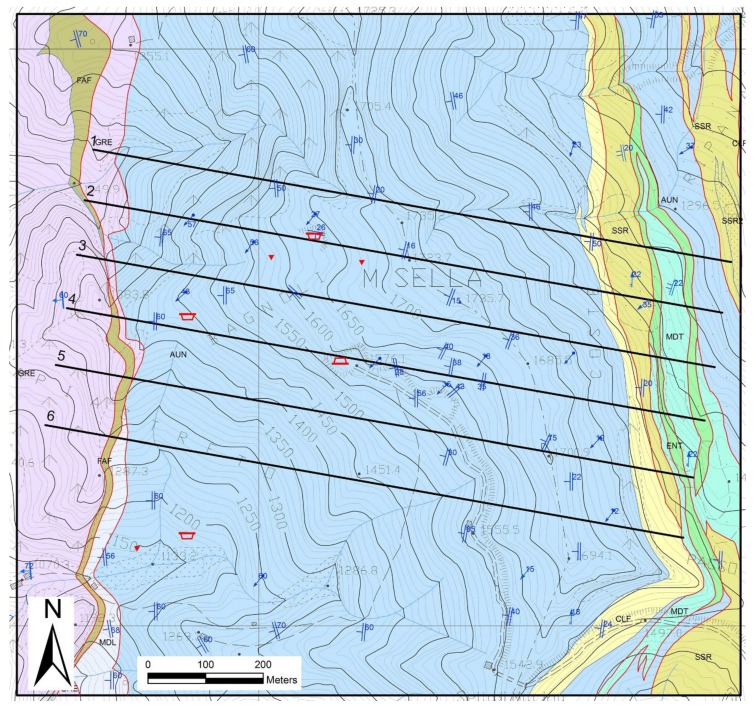
Geological map of the Piastreta underground quarry and surrounding areas. Geological Formation acronyms: FAF: “Filladi Inferiori” (phyllites); GRE: “Grezzoni” (dolomites); MDL: dolomitic marble; AUN: Marble; CLF: Cherty metalimestones; MDT: Metaradiolarites; ENT: Entrochi cherty metalimestones; SSR: Sericitic schists; SSR2: “Cipollini Marbles” (calcschists). Traces of the six geological cross-sections utilized to build the 3D model of Figure 4 are shown.

The litho-stratigraphic sequence includes a Paleozoic basement covered unconformably by an Upper Triassic-Oligocene meta-sedimentary sequence (Figure 2). The Mesozoic cover is represented by thin Triassic continental to shallow water Verrucano-like deposits, overlain by Upper Triassic-Liassic carbonate platform meta-sediments that include dolomites (“Grezzoni”), dolomitic marbles and marbles (worldwide known as “Carrara marbles”). These, in turn, are superimposed by Upper Liassic-Lower Createceous cherty meta-limestones, cherts and calcschists, and by Lower Cretaceous-Lower Oligocene sericitic phyllites and calcschists, with marble interlayers, which are related to deep water sedimentation during drowning of the former carbonate platform. Oligocene sedimentation of turbiditic sandstones (“Pseudomacigno”) finalizes the sedimentary history of the domain [35].

**Figure 3:**
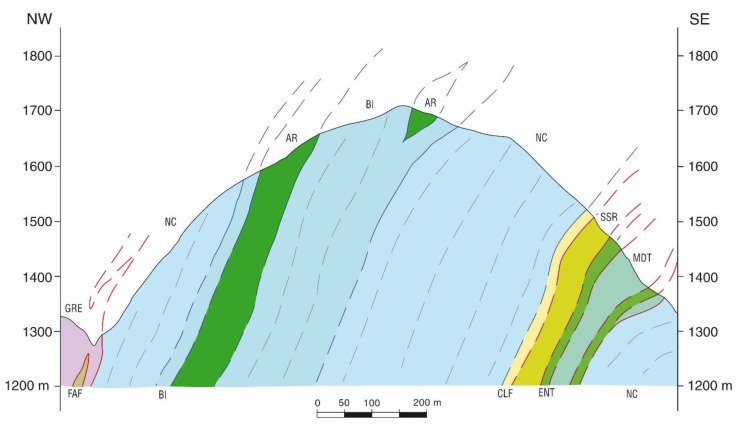
Geological cross-section n. 3 of Figure 2 passing nearby the Piastreta quarry and surrounding area; the section shows also some marketable marble varieties (i.e., BI: “Bianco Piastreta”; AR: “Arabescato Piastreta” and “Brouillè”; NC: undetermined/unextracted marble).

**Figure 4:**
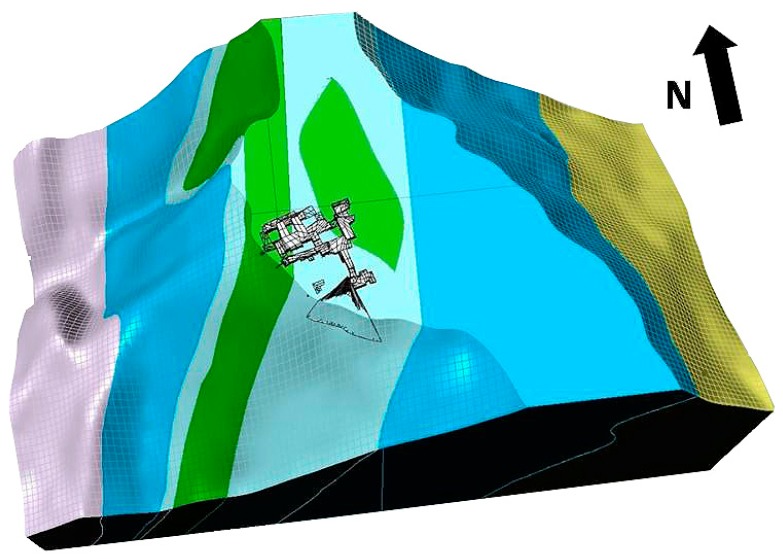
Perspective view of the 3D geological model. Colors of the geological formations coincide with those used for the geological map of Figure 3. Given the low thickness and the purposes of the present research, the model does not include the “Filladi Inferiori” (phyllites) Formation outcropping at the nucleus of the “Monte Tambura” antiform and exposed on the western side of the Sella Mount. Yellow volume of the eastern side refers to the ensemble of the following Formations: Cherty meta-limestone, Metaradiolarites, Entrochi cherty metalimestones, and Sericitic schists.

Figure 3 and Figure 4 show a geological cross-section of the Piastreta quarry area and a perspective view of the created 3D geological model, respectively.

The Apuan Alps geological setting results from two main tectono-metamorphic events: a ductile compressional event D_1_ (Lower Miocene), due to the continental collision between Sardinia-Corsica block and Adria plate; a subsequent extensional D_2_ event (Upper Miocene) leading to an isostatic rebalance [36].

The marble extraction of the Piastreta quarry is executed in tunnel within a D_1_ geological structure known as the "Monte Tambura" antiform. The quarry is located in the inverted limb of the antiform, an E-NE vergent fold located in the central area of the Apuan Alps, with an amplitude of about 15 km and a general N-S strike (Figure 1). The marketable marble varieties of the Piastreta quarry are represented by the “Bianco Piastreta”, belonging to the group of white marbles, the “Arabescato Piastreta” and “Calacatta Piastreta” referable to the breccia marbles group. “Bianco Piastreta” is a white marble characterized by a middle/fine size grain. “Arabescato Piastreta” is a clast supported meta-breccia with various sizes marble elements in matrix that is variable in color from grey to dark green. In some areas of the quarry the clasts of meta-breccia are typically white in color; this type of marble is marketed under the name “Brouillè” for its particular similarity with the “Arabescato Brouillè” extracted in the near Carrara basin.

“Calacatta Piastreta” is a breccia marble containing centimetric to decimetric elements in a matrix that is variable in color from yellow to grey-green with opaque minerals and sub-millimetric pyrite.

## 3. Materials and Methods

### 3.1. Data Acquisition and Processing

The Piastreta quarry was object of TLS activities on June 2016 and June 2017. The device used for this study is a TX 8 instrument (Trimble^TM^, Sunnyvale, CA, USA) characterized by an operating wavelength of 1500 nm and a range of 120 to 340 m, depending on target reflectivity. The system produces 1 million pts/sec, with a range noise lower than 2 mm from 2 m to 120 m distances. It has a 360° horizontal field of view, 310° in vertical, and it must be firmly mounted on a tripod. The whole underground quarry was observed from different viewpoints in order to eliminate any possible shadow zone. A great number of acquisition points were necessary: namely 24 stations in the first measurement campaign, necessary to cover the entire site, and 17 in the second one; the lower number of scan stations was because particular attention was reserved only in changed areas over one year of excavation activities. A point spacing of 11.3 mm at a distance of 30 m was selected with a maximum range of 120 m. These scan parameters were chosen in consideration of the device characteristics, distance between sensor and targets, beam divergence, shape and reflectivity of the marble walls, and, finally, environmental conditions. Taking into account that the width of the quarry tunnels is on average 10 m, TLS was generally placed at the center of the quarry tunnels in such a way that targets were acquired at a distance of about 5 m, resulting in a point spacing of about 1.8 mm. Adding to that the great number of TLS stations, the obtained point cloud density resulted to be appropriate for the purposes of the study. After each scanning, the device was replaced by a D7100 digital camera (Nikon^TM^, Melville, NY, USA) to provide RGB colored photos with the goal of acquiring co-axial images for point cloud texturing. 

**Figure 5:**
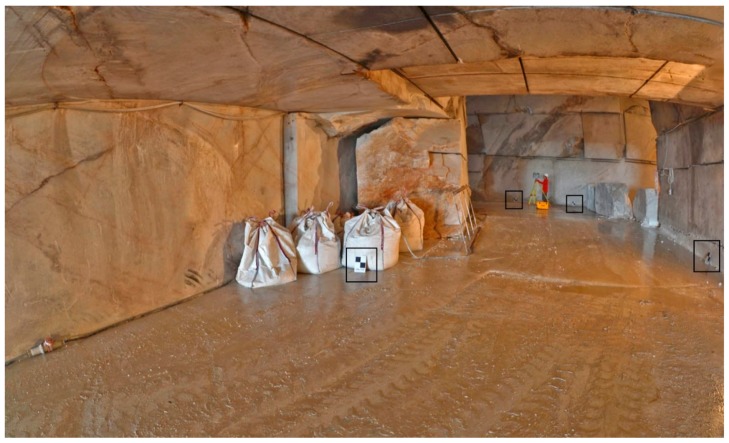
Operational phases of TLS. Example of black and white flat targets (highlighted with black rectangles) placed on the scene for TLS dataset georeferencing.

Moreover, in order to define a permanent World Geodetic System (WGS) for future multitemporal acquisitions and other possible purposes, five supports for High Definition Surveying (HDS) targets were permanently placed on the rock walls in sites not involved in present and future excavations. Absolute positions of these targets were measured by a Total Station (TS) survey associated with static GNSS survey; the latter was executed out of the quarry entrance and it included the absolute coordinates measurement of two points: a first point was used as origin of the topographic survey, while the second as 0-Azimuth direction for TS orientation. In order to correctly georeference the entire TLS dataset, a series of black and white flat targets was placed on the scene (Figure 5). These targets were included in the TLS survey and measured by the TS. In lab., individual scans of the same epoch were registered together using the Iterative Closest Point (ICP) algorithm [37,38] of Trimble^TM^ Realworks software with the aim of providing a global 3D point cloud. Multitemporal TLS datasets were georeferenced using the topographic info coming from GNSS and TS surveys, then coregistered in order to minimize their relative distances; this last step was necessary in order to properly perform the change detection analysis and it was done by using the “cloud-based registration” tool of Trimble^TM^ Realworks. A data cleaning was also necessary for removing machines, workers and tools present in the scene at the moment of data acquisition. The 2016-point cloud was used as reference, while the 2017-point cloud was matched onto stable areas of the reference point cloud using the ICP algorithm. Stable areas are represented by walls and fronts not subject to excavation activities during the analyzed time span (one year).

### 3.2. Multitemporal Point Clouds Comparison and Volume of Extracted Material Computation

Coregistered point clouds referred to the years 2016 and 2017 were processed using the M3C2 plugin of CloudCompare open source software [39]. The selected approach consists in performing a direct comparison of 3D point clouds without meshing or gridding. The distance between point clouds along their normal surface direction is calculated allowing to assess morphological 3D changes in surface orientation. The software calculates for each distance measurement a confidence interval depending on point cloud roughness and coregistration error [7]. In the coregistration error field, the mutual misalignment between multitemporal point clouds was selected as input data.

Important parameters to be defined for computing distances between multitemporal point clouds are:

- The normal scale (D), the projection scale (d), and the cylinder depth. “D” corresponds to the diameter of a disk centered in a given core point “i”. The disk comprises neighbor points, utilized to automatically fit a plane, from which a normal vector is defined [7]. A uniform normal scale, or a range of normal scales, can be iteratively applied to the considered point cloud. The orientation of the normal (i.e., vertical or horizontal) can be also selected. “d” is the diameter of a cylinder which intersects the clouds under comparison and, going through a given point “i”, it has the major axis oriented along the normal vector. The cylinder separates two subsets from the clouds, whose mean distribution gives their average positions, i_1_ and i_2_. In this method, the local distance between the point clouds corresponds to the length of the major axis segment between i_1_ and i_2_. The length standard deviation estimates the point cloud roughness along the normal direction. The max cylinder depth corresponds to the magnitude of difference between the compared point clouds.
- The core points to be utilized for measuring the distances. The main idea is that while TLS clouds are generally very dense, it is not necessary to measure the distance at such a high density and a sub-sampled version of the entire cloud can be used to speed up the computation [39,40].
- The coregistration error (*coreg*) which is related to the accuracy of multitemporal point clouds alignment.

As already mentioned, in this work the 2016-point cloud was set as reference and the 2017 as compared cloud. In order to speed up the computation, the core points refer to a sub-sampled version of the input cloud at a resolution of 0.1 m. After several tests, a fixed normal scale of 0.80 m, a projection scale of 0.80 m and a maximum depth of 5 m were chosen. Moreover, it was verified if the chosen “D” and “d” scales respected the suggestions of Lague et al. [7]; precisely, it was verified whether the D scale was 20–25 times the estimated roughness for the two point clouds, and if the “d” scale, given the point cloud density, was big enough to have at least 4 points included.

The cloud-based registration tool of Trimble^TM^ Realworks gives the percentage of coincident points and the “overall cloud-to-cloud error”; the latter was used as coregistration error in the M3C2 plugin. As output, M3C2 computes a new cloud comprising the following information associated to each point: distance to the closest corresponding point, distance uncertainty and significant change. The distance uncertainty corresponds to the confidence interval, also referred as Level of Detection at 95% (LOD_95%_), and it is calculated by the following equation (e.g., [41]):

(1) $${\mathrm{LOD}}_{95\%}=\pm 1.96\left(\sqrt{\frac{\sigma_{1}{\left(d\right)}^{2}}{n_{1}}+\frac{\sigma_{2}{\left(d\right)}^{2}}{n_{2}}}+\;\mathrm{coreg}\right)$$

where: *d* is the projection scale, $\sigma_{1}{\left(d\right)}^{2}$ and $\sigma_{2}{\left(d\right)}^{2}$ are the local roughness of the point clouds *n*_1_ and *n*_2_. In this way, the spatially variable confidence interval is calculated and the statistically significant change is provided: a distance is considered statistically significant when is greater than the LOD_95%_. This relation was verified for each point of the two compared point clouds. The distance computation with M3C2 plugin was performed for the whole extent of the point clouds, while the plugin efficiency was assessed in unchanged zones of the area.

For the computation of extracted material volumes, meshes were generated by Poisson Surface Reconstruction plugin proposed by Kazhdan et al. [31] in areas of evident change detection. The CloudCompare software (version 2.9.1) was used for this step of the analysis. In order to correctly use this plugin, normals of the point clouds were computed and cleaned (i.e., the orientation of all normals must be correct/consistent and not too noisy). By default, the algorithm must be applied on closed 3D shapes. In order to perform this task, multitemporal point clouds corresponding to excavated areas were segmented. The 2017-point cloud at the top and 2016-point cloud at the bottom were merged in order to obtain closed 3D shapes. The volume computation of the 3D mesh objects was performed within CloudCompare software in order to quantify the extracted material over one year of quarrying activity.

## 4. Results

The registration and georeferencing errors of multitemporal TLS datasets, plus the coregistration error between the 2016- and 2017-point clouds, are provided in Table 1. TLS point clouds have an accuracy of a few millimeters with a mutual misalignment of 3.5 cm. The obtained coregistration error was included in the computation of the multitemporal distance confidence.

**Figure 6:**
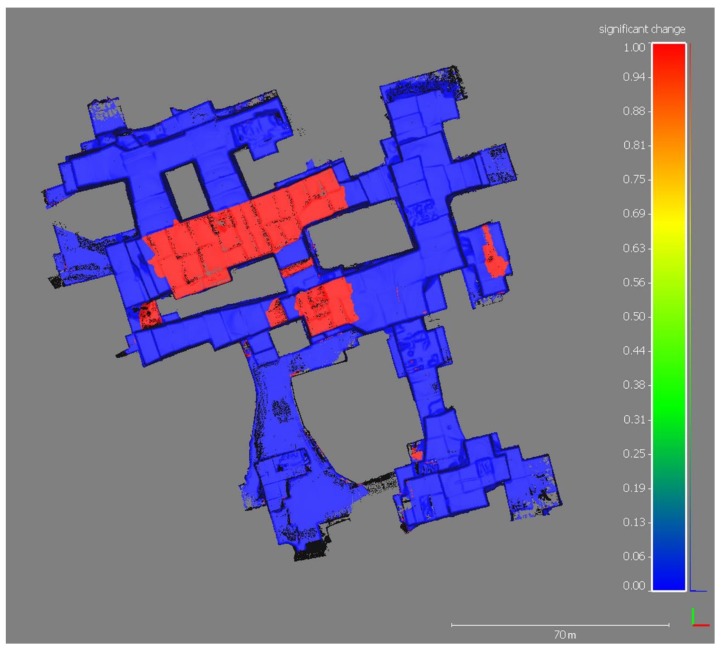
Map of significant changes. Red values, equal to 1, indicate areas of significant variations contrary to blue values, equal to 0, that represent no-change.

**Figure 7:**
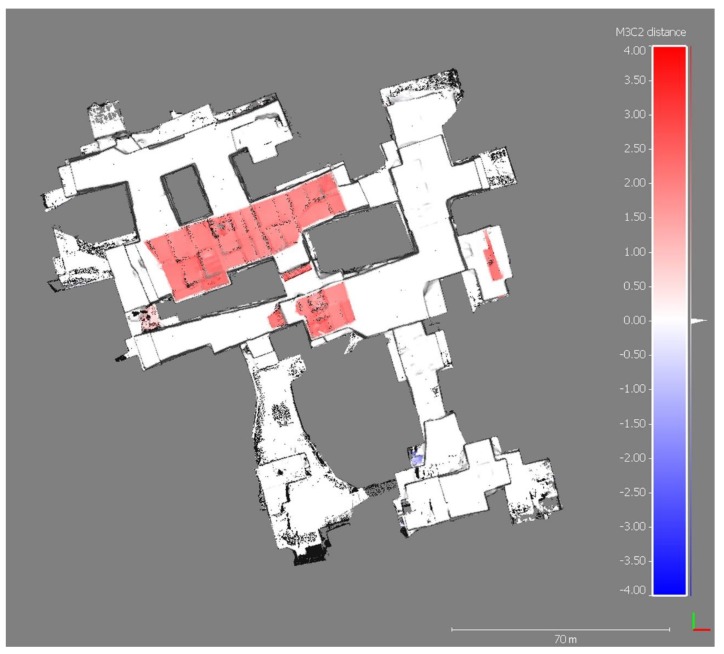
Map of computed distance to the closest corresponding point (the measurement unit of the color bar is meter).

**Figure 8:**
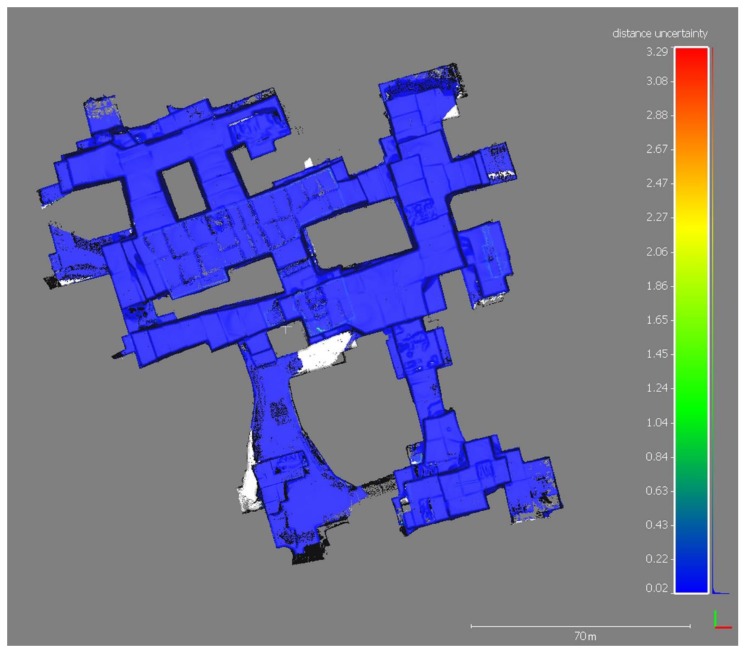
Map of distance uncertainty between corresponding points (the measurement unit of the color bar is meter).

The map of significant changes (Figure 6) shows the morphological variations between the years 2016 and 2017; non-significant changes correspond to stable zones (blue areas in Figure 6). Figure 7 shows the distance between the two point clouds, while the distance uncertainty map is displayed in Figure 8.

**Figure 9:**
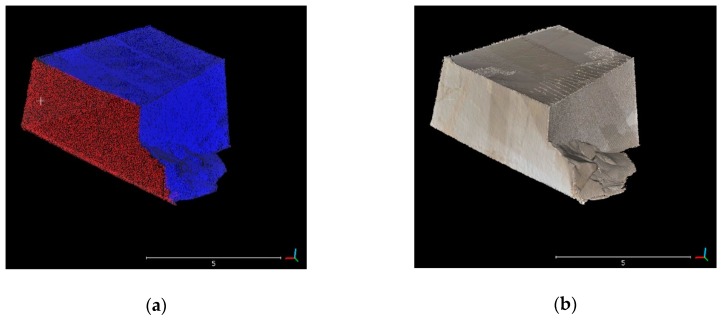
(**a**) Closed 3D shape made by the 2017-point cloud at the top (blue color) and the 2016-point cloud at the bottom (red color); (**b**) 3D textured model (mesh).

Changed areas, representing the material extracted during the analyzed time span, were segmented and merged in order to obtain closed 3D shapes. Meshes were made from segmented 3D point clouds (Figure 9a). Figure 9b shows the 3D textured model obtained by associating the mesh of a rocky block with the photos provided by the digital camera. The volume of single extraction areas over one year of quarrying activity was calculated and results are shown in Table 2; the total amount of extracted material resulted equal to 4707.5 m^3^ ± 4%. The estimation of errors in volume computation of the extracted material was calculated for each mining zone by multiplying the surface area of each extraction site by the coregistration error between the TLS datasets. The volume computation errors for some extraction site (i.e., V1, V2, Vn) are provided in Table 2.

**Figure 10:**
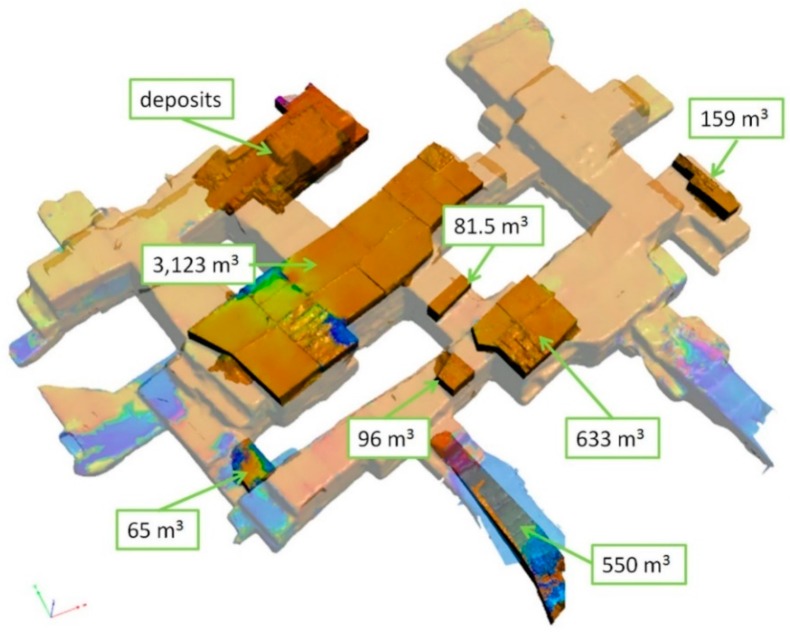
Overall Piastreta 3D model with highlighted the single representations of extracted marble blocks (individual volumes are expressed in m^3^).

Finally, in order to display a visual summary of the extracted material, a digital multitemporal 3D map was created in PDF format (Figure 10) by using 3D Systems Geomagic Studio software. In this file the sites of marble extraction within the Piastreta quarry are represented by individual 3D models with associated relative volumes in cubic meter (m^3^).

## 5. Discussion

The change detection analysis of the Piastreta quarry allowed to obtain an understanding of geomorphic change detection during one-year time span of marble excavation. The experience carried out has confirmed as TLS technology can be used for this kind of application with rapid times of execution (one-day survey) and accuracy (mm). In fact, the main advantages in comparison with the traditional topographic surveying are the size and completeness of the area surveyed in a very short time, and the very high-level of detail, without having to choose a very limited set of points to be measured. Nevertheless, the achieved accuracy in volume computation of extracted marble blocks depends on several factors. The user experience and the foresight in executing the field survey and data processing play an important role. Among the others, the accuracies of registration and coregistration steps are related to the care of point cloud cleaning. Multitemporal point clouds must be properly coregistered in order to minimize the distance between corresponding surface features. Incompleteness of removal from the point cloud of working tools and machines, as well as wire in the walls, water on the floor, working dust, etc. leaves noise and compromises the subsequent analyses. Moreover, additional factors, such as the attention in filtering procedures and the quality of segmentation and 3D model creation, are important. Lastly, also the geometrical characteristics and shapes of the investigated surfaces and the material properties may influence the quality of outcomes; marble translucency and heterogeneous structure must be taken in account because walls surfaces may produce significant bias and increased noise in the geometric measurements [42]. The combination of all these factors may have had a role in this work: considering that the achieved registration and georeferencing errors are at millimeter level, and that the coregistration between 2016- and 2017-point clouds was performed on stable areas of the quarry, the reasons given above may have determined a coregistration error of one order of magnitude higher than the previous ones. However, the achieved coregistration accuracy can be considered sufficient for the study aim of computing the volume of extracted material over one year of quarrying activities. The M3C2 plugin of CloudCompare open source software allowed to obtain a satisfying change detection result starting from the two point clouds. Its use has shown a series of advantages compared to more diffuse techniques (e.g., DoD). The algorithm does not require gridding or meshing of the point cloud, hence uncertainties in grid elevation can be avoided. The M3C2 algorithm works in 3D and, in case of missing data, it does not compute false distances along the normal direction to avoid errors. The estimation of the spatially variable confidence interval is a great further advantage because it allows to estimate the accuracy of local distance measurements. Lague et al. [7] define the confidence interval as the minimum detectable change at 95% confidence level (i.e., LOD_95%_) and it can be used to assess if a detected change is statistically significant or not. The M3C2 algorithm verifies such relation automatically for each point, and assesses if the distances fall beneath the confidence interval boundary (“non-significant change” in Figure 6) or above (“significant change”). In our case study, among the 1,634,948 points that constitute the M3C2-derived cloud with project core points on 2017-point cloud, 1,369,897 were automatically classified as not relevant for change detection. The obtained average distance uncertainty, equal to 0.04 m with a standard deviation of 0.06 m, can be considered suitable given the TLS technique used in this study, the spatial resolution of acquisition, and the presence of noisy features in the scenes (e.g., water, working dust, manpower and machines in movement during data acquisition).

The digital multitemporal map of the quarry, shown in Figure 10, is an example of a 3D digital cartographic product which can be obtained from change detection analysis like that described in this study. This deliverable summarizes changes of the active underground extent through time representing a fundamental tool to support the mining activity planning and control. In order to produce it, changed areas were segmented and 3D models were created from the point clouds.

## 6. Conclusions

Underground marble quarries of the Apuan Alps are nowadays dynamic environments characterized by rapid and large morphological changes thanks to mechanical-technological advances. For this reason, monitoring of quarrying activities and geomorphic changes through time plays a fundamental role for work planning in respect of safety and productivity.

Traditional surveying with total station can be labor intensive and time consuming (thus also expensive). TLS constitutes an ideal technique for modelling the excavation surfaces generating dense 3D point clouds, rapidly providing accurate and detailed geometric info. Surveying and assessment of morphological changes in underground marble quarries is successfully possible by the terrestrial laser scanning method as supported by this case study. Moreover, point clouds can be treated using open source software. 3D change detection analysis can be performed on point clouds related to different time periods. Surfaces can be created from point clouds and closed 3D shapes volume calculated. Furthermore, TLS allows quarrymen to gain information about the geomorphic differences of extracted material and possible unstable zones.

Finally, it is necessary to underline the importance of validating the obtained results for studies aimed to the volumetric estimation of the extracted material, like the one presented in this paper; a careful ground truthing of excavated volumes is always necessary for scientists and technicians engaged in multitemporal change detection analysis. For example, “Structure from Motion” (SfM) photogrammetry is nowadays considered an alternative and reliable method for collecting data and to derive dense 3D point clouds [3,24,28,30]. Fast data acquisition, point density and high accuracy are common big advantages of both, TLS and photogrammetry, if compared to classical survey measurements. Multitemporal 3D point clouds obtained from these methodologies may be analyzed and volume calculations derived. In this case study, volumetric results were validated by traditional topographic surveys carried out by the technicians of the Piastreta marble quarry, and by functionaries of Massa Municipality, whose production records were kindly available for comparison but not for publication.

## Figures and Tables

**Table 1:** Characteristics of the TLS datasets used for multitemporal marble extraction assessment.

TLS Dataset	N° Points	Registration Error (m)	Georeferencing Error (m)	Coregistration Error (m)
Piastreta 2016	7,141,309	0.005	0.004	0.035
Piastreta 2017	4,107,555	0.009	0.003	

**Table 2:** Example of extracted material estimation in the analyzed time span.

	V1	V2	V3	V4	V5	V6	V7
**Volume (m^3^)**	550	633	81.5	159	65	3123	96
**Surface area (m^2^)**	772	720	133	288	146	2934	150
**Error estimation (%)**	5.05	4.09	5.87	6.52	8.08	3.38	5.62

## References

[1] Fanti R., Gigli G., Lombardi L., Tapete D., Canuti P. (2013). Terrestrial laser scanning for rockfall stability analysis in the cultural heritage site of Pitigliano (Italy). Landslides.

[2] Abellán A., Oppikofer T., Jaboyedoff M., Rosser N.J., Lim M., Lato M.J. (2014). Terrestrial laser scanning of rock slope instabilities. Earth Surf. Process. Landforms.

[3] Assali P., Grussenmeyer P., Villemin T., Pollet N., Viguier F. (2014). Surveying and modeling of rock discontinuities by terrestrial laser scanning and photogrammetry: Semi-automatic approaches for linear outcrop inspection. J. Struct. Geol..

[4] O’Neal M.A., Pizzuto J.E. (2011). The rates and spatial patterns of annual riverbank erosion revealed through terrestrial laser-scanner surveys of the South River, Virginia. Earth Surf. Process. Landforms.

[5] Brasington J., Vericat D., Rychkov I. (2012). Modeling river bed morphology, roughness, and surface sedimentology using high resolution terrestrial laser scanning. Water Resour. Res..

[6] Day S.S., Gran K.B., Belmont P., Wawrzyniec T. (2013). Measuring bluff erosion part 1: Terrestrial laser scanning methods for change detection. Earth Surf. Process. Landforms.

[7] Lague D., Brodu N., Leroux J. (2013). Accurate 3D comparison of complex topography with terrestrial laser scanner: Application to the Rangitikei canyon (N-Z). ISPRS J. Photogramm. Remote Sens..

[8] Kuhn D., Prüfer S. (2014). Coastal cliff monitoring and analysis of mass wasting processes with the application of terrestrial laser scanning: A case study of Rügen, Germany. Geomorphology.

[9] Kovanič Ľ., Blišťan P. (2014). Quarry Wall Stability Assessment Using TLS Method. Adv. Mater. Res..

[10] Deliormanli A.H., Maerz N.H., Otoo J. (2014). Using terrestrial 3D laser scanning and optical methods to determine orientations of discontinuities at a granite quarry. Int. J. Rock Mech. Min. Sci..

[11] Havaej M., Coggan J., Stead D., Elmo D. (2016). A combined remote sensing–numerical modelling approach to the stability analysis of delabole slate quarry, Cornwall, UK. Rock Mech. Rock Eng..

[12] Campbell A.D., Thurley M.J. (2017). Application of laser scanning to measure fragmentation in underground mines. Min. Technol..

[13] Long N.Q., Buczek M.M., Szlapińska S.A., Nam B.X., Nghia N.V., Cuong C.X. (2018). Accuracy assessment of mine walls’ surface models derived from terrestrial laser scanning. Int. J. Coal Sci. Technol..

[14] Živec T., Anžur A., Verbovšek T. (2018). Determination of rock type and moisture content in flysch using TLS intensity in the Elerji quarry (south-west Slovenia). Bull. Eng. Geol. Environ..

[15] Lerma García J.L., Van Genechten B., Heine E., Santana Quintero M. (2008). Theory and practice on Terrestrial Laser Scanning.

[16] Vosselman G., Hans-Gerd M. (2010). Airborne and Terrestrial Laser Scanning.

[17] James L.A., Hodgson M.E., Ghoshal S., Latiolais M.M. (2012). Geomorphic change detection using historic maps and DEM differencing: The temporal dimension of geospatial analysis. Geomorphology.

[18] Bossi G., Cavalli M., Crema S., Frigerio S., Quan Luna B., Mantovani M., Marcato G., Schenato L., Pasuto A. (2015). Multi-temporal LiDAR-DTMs as a tool for modelling a complex landslide: A case study in the Rotolon catchment (eastern Italian Alps). Nat. Hazards Earth Syst. Sci..

[19] Schürch P., Densmore A.L., Rosser N.J., McArdell B.W. (2011). Dynamic controls on erosion and deposition on debris-flow fans. Geology.

[20] Rychkov I., Brasington J., Vericat D. (2012). Computational and methodological aspects of terrestrial surface analysis based on point clouds. Comput. Geosci..

[21] Barbarella M., Fiani M., Lugli A. (2015). Landslide monitoring using multitemporal terrestrial laser scanning for ground displacement analysis. Geomatic. Nat. Hazards Risk.

[22] Abellan A., Derron M.-H., Jaboyedoff M. (2016). “Use of 3D Point Clouds in Geohazards” Special Issue: Current Challenges and Future Trends. Remote Sens..

[23] Al-Rawabdeh A., Moussa A., Foroutan M., El-Sheimy N., Habib A. (2017). Time series UAV image-based point clouds for landslide progression evaluation applications. Sensors.

[24] Esposito G., Salvini R., Matano F., Sacchi M., Danzi M., Somma R., Troise C. (2017). Multitemporal monitoring of a coastal landslide through SfM-derived point cloud comparison. Photogramm. Rec..

[25] Watson C.S., Quincey D.J., Smith M.W., Carrivick J.L., Rowan A.V., James M.R. (2017). Quantifying ice cliff evolution with multi-temporal point clouds on the debris-covered Khumbu Glacier, Nepal. J. Glaciol..

[26] Keilig K.-P., Dietrich A., Krautblatter M., Shakoor A., Cato K. (2018). Comparison of Multi-temporal Elevation Models of a Debris-Flow Channel. IAEG/AEG Annual Meeting Proceedings, San Francisco, CA, USA.

[27] Mayr A., Rutzinger M., Geitner C. (2018). Multitemporal analysis of objects in 3D point clouds for landslide monitoring. Int. Arch. Photogramm. Remote Sens. Spat. Inf. Sci..

[28] Nikolakopoulos K.G., Antonakakis A., Kyriou A., Koukouvelas I., Stefanopoulos P. (2018). Comparison of terrestrial laser scanning and structure-from-motion photogrammetry for steep slope mapping. Earth Resources and Environmental Remote Sensing/GIS Applications IX.

[29] Rossi G., Tanteri L., Tofani V., Vannocci P., Moretti S., Casagli N. (2018). Multitemporal UAV surveys for landslide mapping and characterization. Landslides.

[30] Stumpf A., Malet J.P., Allemand P., Pierrot-Deseilligny M., Skupinski G. (2015). Ground-based multi-view photogrammetry for the monitoring of landslide deformation and erosion. Geomorphology.

[31] Kazhdan M., Bolitho M., Hoppe H. Poisson Surface Reconstruction. http://dl.acm.org/citation.cfm?id=1281957.1281965.

[32] Carmignani L., Kligfield R. (1990). Crustal extension in the northern Apennines: The transition from compression to extension in the Alpi Apuane Core Complex. Tectonics.

[33] Elter P. (1975). Introduction a la geologie de l’Apennin septentrional. Bulletin de la Société géologique de France.

[34] Molli G., Siegesmund S., Fügenschuh B., Froidzheim N. (2008). Northern Appennine-Corsica Orogenic System: An Updated Review.

[35] Conti P., Carmigani L., Giglia G.M.M., Fantozzi P.L., Morini D., Bruni P. (2004). Evolution of geological interpretations in the Alpi Apuane Metamorphic Complex, and their relevance for the geology of the Northern Apennines. The Regione Toscana Project of Geological Mapping.

[36] Molli G., Vaselli L., Mazzoli S., Buler V. (2006). Structures, interference patterns and strain regime during mid-crustal deformation in the Alpi Apuane (Northern Appennines, Italy). Styles of Continental Contraction.

[37] Yang C., Medioni G. (1992). Object modelling by registration of multiple range images. Image Vis. Comput..

[38] Besl P.J., McKay N.D. (1992). A Method for Registration of 3-D Shapes. IEEE Trans. Pattern Anal. Mach. Intell..

[39] CloudCompare Official Web Site. http://www.danielgm.net/cc/.

[40] Brodu N., Lague D. (2012). 3D terrestrial lidar data classification of complex natural scenes using a multi-scale dimensionality criterion: applications in geomorphology. ISPRS J. Photogramm. Remote Sens..

[41] Borradaile G. (2003). Statistics of Earth Science Data: Their Distribution in Time, Space and Orientation.

[42] Godin G., Rioux M., Levoy M., Cournoyer L., Blais F. An assessment of laser range measurement on marble surfaces. Proceedings of the 5th Conference on Optical 3D Measurement Techniques.

